# *Astragalus membranaceus* Improves Exercise Performance and Ameliorates Exercise-Induced Fatigue in Trained Mice

**DOI:** 10.3390/molecules19032793

**Published:** 2014-03-03

**Authors:** Tzu-Shao Yeh, Hsiao-Li Chuang, Wen-Ching Huang, Yi-Ming Chen, Chi-Chang Huang, Mei-Chich Hsu

**Affiliations:** 1 School of Nutrition and Health Sciences, Taipei Medical University, Taipei 11031, Taiwan; 2 National Laboratory Animal Center, National Applied Research Laboratories, Taipei 11529, Taiwan; 3 Graduate Institute of Athletics and Coaching Science, National Taiwan Sport University, Taoyuan 33301, Taiwan; 4 Graduate Institute of Sports Science, National Taiwan Sport University, Taoyuan 33301, Taiwan; 5 Department of Sports Medicine, Kaohsiung Medical University, Kaohsiung 80708, Taiwan

**Keywords:** Huangqi, exhaustion exercise, lactate, ammonia, glycogen

## Abstract

*Astragalus membranaceus* (AM) is a popular “Qi-tonifying” herb with a long history of use as a Traditional Chinese Medicine with multiple biological functions. However, evidence for the effects of AM on exercise performance and physical fatigue is limited. We evaluated the potential beneficial effects of AM on ergogenic and anti-fatigue functions following physiological challenge. Male ICR strain mice were randomly assigned to four groups (*n* = 10 per group) for treatment: (1) sedentary control and vehicle treatment (vehicle control); (2) exercise training with vehicle treatment (exercise control); and (3) exercise training with AM treatment at 0.615 g/kg/day (Ex-AM1) or (4) 3.075 g/kg/day (Ex-AM5). Both the vehicle and AM were orally administered for 6 weeks. Exercise performance and anti-fatigue function were evaluated by forelimb grip strength, exhaustive swimming time, and levels of serum lactate, ammonia, glucose, and creatine kinase after 15-min swimming exercise. Exercise training combined with AM supplementation increased endurance exercise capacity and increased hepatic and muscle glycogen content. AM reduced exercise-induced accumulation of the byproducts blood lactate and ammonia with acute exercise challenge. Moreover, we found no deleterious effects from AM treatment. Therefore, AM supplementation improved exercise performance and had anti-fatigue effects in mice. It may be an effective ergogenic aid in exercise training.

## 1. Introduction

*Astragalus membranaceus* (AM) is a well-known the “Qi-tonifying” or adaptogenic herb used in Traditional Chinese Medicine. It has been prescribed for centuries for general debilitation and chronic illnesses and to increase overall vitality [1]. The main constituents of AM roots are polysaccharides, saponins, flavonoids, amino acids and trace elements [2,3]. More than 40 constituents in *Astragalus* saponins have been identified from the *Astragalus* root [4]. Astragalosides have various pharmacological activities and are used as a quality-control marker of AM [5]. In recent decades, molecular features and pharmacokinetics and pharmacological actions of Astragaloside polysaccharides and other active ingredients from the plant have been studied extensively, which could enable its clinical use [6]. AM has effects against myocardial damage [7,8,9,10], promotes angiogenesis [11,12] and protects endothelial function [13], prevents ultraviolet A-induced photoaging [14], is hepatoprotective [15], improves insulin resistance [16] and is involved in immunomodulatory activities [17,18,19]. However, few studies have directly addressed the possible anti-fatigue function of AM.

Fatigue is a complex phenomenon that can be defined as the inability to maintain expected muscle strength, leading to reduced performance during prolonged exercise and has classified physical and/or mental fatigue [20]. Physical fatigue is also called peripheral fatigue, which can be derived from the action of the muscles and may be accompanied by deterioration in functional performance [21,22,23]. Chronic fatigue can lead to severe health problems [24,25]. There are at least two mechanisms that can explain the occurrence of physical fatigue: oxidative stress and energy exhaustion [26]. Overloading work or exhaustive exercise can lead to the accumulation of excess reactive free radicals, which result in tissue damage. Exhaustion theory suggests that energy source depletion and excess metabolite accumulation lead to fatigue [27]. However, several studies have shown that exogenous nutrition supplement can reduce exercise-induced physical fatigue [28,29,30,31,32]. Research into specific nutrients or herbal supplements is needed to find agents that reduce metabolite production and/or improve energy utilization.

Herbal medicines and food factors have been investigated as an important resource for postponing fatigue, accelerating the elimination of fatigue-related metabolites, and improving exercise performance. AM has long been considered a potent remedy for regulating the body balance, with few adverse effects, but scientific evidence of its action is lacking. AM may be an anti-fatigue herbal supplement candidate. Here, we examined whether AM supplementation before intensive aerobic exercise training could change body composition, physical activities, and physiologic features *in vivo* in mice.

## 2. Results and Discussion

### 2.1. Body Weight and Other Metabolism-Related Organ Weights

Morphological data from each experimental group are listed in Table 1. Initial and final *body* weights did not differ among treatment groups. The food intake was higher by 1.12-, 1.10 and 1.05-fold (*p* < 0.05), respectively, for the exercise control, Ex-AM1 and Ex-AM5 groups, than the vehicle control group. The groups did not differ in weights of liver, kidney, epididymal fat pad, muscle, and brown adipose tissue. The relative tissue weight (%) is a measure of different tissue weights adjusted by individual body weight, and relative liver weight was lower for the Ex-AM5 group than the vehicle group (*p* < 0.05).

**Table 1:** General characteristics of the experimental groups.

Characteristic	Vehicle Control	Exercise Control	Ex-AM1	Ex-AM5
Initial body weight (g)	25.43 ± 0.24	25.47 ± 0.20	25.53 ± 0.29	25.49 ± 0.28
1 week body weight (g)	29.94 ± 0.57	29.11 ± 0.40	28.76 ± 0.50	28.78 ± 0.57
2 week body weight (g)	31.54 ± 0.59	30.07 ± 0.49	31.55 ± 0.50	31.41 ± 0.68
3 week body weight (g)	32.63 ± 0.48	32.43 ± 0.50	32.84 ± 0.48	33.26 ± 0.85
4 week body weight (g)	33.52 ± 0.44	33.53 ± 0.41	33.52 ± 0.53	34.23 ± 0.85
5 week body weight (g)	34.50 ± 0.45	33.68 ± 0.33	34.59 ± 0.56	35.11 ± 0.81
Final body weight (g)	35.59 ± 0.51	35.80 ± 0.46	36.28 ± 0.44	37.18 ± 0.76
Food intake (g/day)	6.78 ± 0.01 ^a^	7.61 ± 0.01 ^d^	7.44 ± 0.03 ^c^	7.11 ± 0.03 ^b^
Food efficiency (%)	1.49 ± 0.06	1.35 ± 0.05	1.44 ± 0.05	1.64 ± 0.08
Liver (g)	2.16 ± 0.03	2.14 ± 0.03	2.05 ± 0.03	2.06 ± 0.04
Kidney (g)	0.61 ± 0.01	0.65 ± 0.02	0.62 ± 0.02	0.63 ± 0.01
Epididymal fat pads (g)	0.52 ± 0.03	0.47 ± 0.02	0.42 ± 0.04	0.46 ± 0.02
Muscle (g)	0.36 ± 0.01	0.36 ± 0.01	0.37 ± 0.01	0.37 ± 0.01
Brown adipose tissue (g)	0.13 ± 0.01	0.15 ± 0.01	0.16 ± 0.01	0.16 ± 0.01
Relative liver weight (%)	6.07 ± 0.07 ^b^	5.97 ± 0.07 ^b^	5.67 ± 0.07^b^	5.54 ± 0.11 ^a^
Relative kidney weight (%)	1.72 ± 0.03	1.81 ± 0.04	1.71 ± 0.04	1.71 ± 0.04
Relative epididymal fat pads weight (%)	1.45 ± 0.06	1.30 ± 0.07	1.17 ± 0.12	1.23 ± 0.07
Relative muscle weight (%)	1.01 ± 0.02	1.02 ± 0.02	1.02 ± 0.02	1.00 ± 0.03
Relative brown adipose tissue weight (%)	0.37 ± 0.01	0.42 ± 0.02	0.43 ± 0.03	0.43 ± 0.03

Data are mean ± SEM for *n* = 10 mice in each group. Data in the same row with different superscript letters (a, b, c and d) differ significantly, *p* < 0.05, by one-way ANOVA. Food efficiency (%): body weight gain (g/day) ÷ food intake (g/day) × 100%. Muscle mass includes both gastrocnemius and soleus muscles in the back part of the lower legs.

### 2.2. Effects of AM on Forelimb Grip Strength

**Figure 1:**
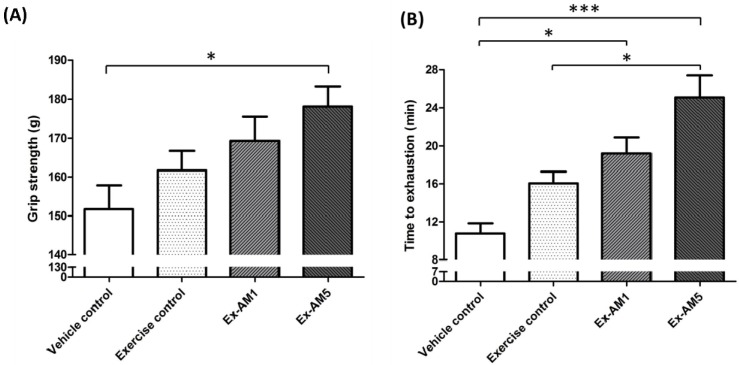
Effect of *A. membranaceus* (AM) supplementation on forelimb grip strength (**A**) and swimming exercise performance (**B**). Data are mean ± SEM of *n* = 10 mice in each group by one-way ANOVA. * *p* < 0.05; *** *p* < 0.001.

The forelimb grip strength of mice increased with Ex-AM5 supplementation than with the vehicle treatment (Figure 1A). On trend analysis, grip strength dose-dependently increased with AM dose during training intervention (*p* < 0.005). Thus, high dose of AM may contribute to physiological activities.

### 2.3. Effect of AM on Exercise Performance in Weight-loaded Swim Test

Exercise endurance is an important variable in evaluating anti-fatigue treatment. Exercise endurance in mice with a swim test increased with Ex-AM1 and Ex-AM5 supplementation than with the vehicle treatment (Figure 1B). At higher AM doses, exercise performance was significantly longer, by 2.33-fold (*p* < 0.05), with Ex-AM5 compared to exercise control. On trend analysis, exercise performance dose-dependently increased with AM dose (*p* < 0.005). Therefore, exercise training combined with AM supplementation significantly increased exercise performance.

### 2.4. Effect of Exercise Training Combined with AM Supplementation on the Serum Levels of Lactate, Ammonia, Glucose and Creatine Kinase (CK) After Acute Exercise Challenge

**Figure 2:**
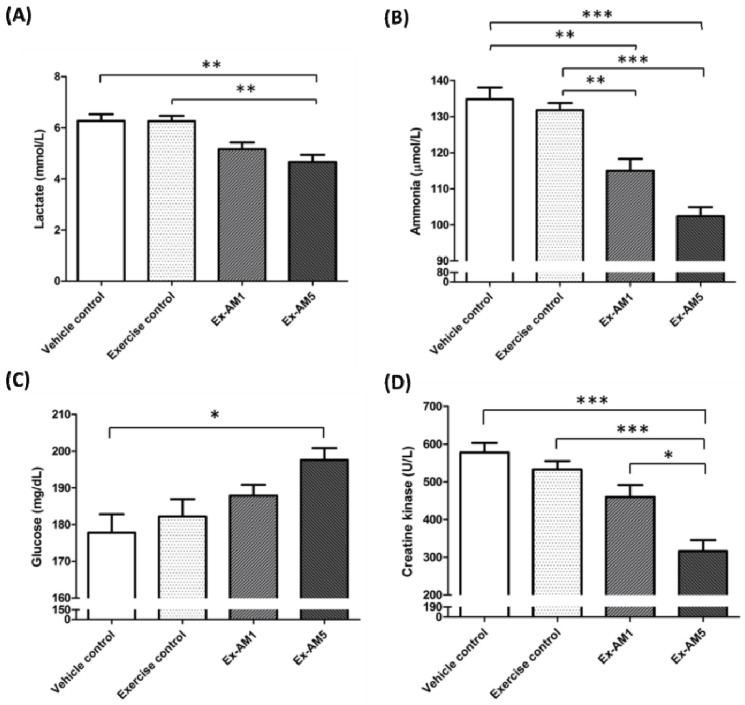
Effect of AM supplementation on serum lactate (**A**), ammonia (**B**), glucose (**C**), and CK (**D**) levels after a 15-min swim test without weight loading. Data are mean ± SEM of *n* = 10 mice in each group by one-way ANOVA. Different letters indicate a significant difference *p* value. * *p* < 0.05; ** *p* < 0.01; *** *p* < 0.001.

Muscle fatigue after exercise can be evaluated by biochemical indicators including lactate, ammonia, glucose and CK levels after exercise [30]. Lactate levels decreased with Ex-AM5 supplementation than with the vehicle or exercise only treatment (Figure 2A). Serum ammonia levels decreased with Ex-AM1 and Ex-AM5 supplementation than with the vehicle or exercise only treatment (Figure 2B). Serum glucose contents increased with Ex-AM5 supplementation than with the vehicle treatment (Figure 2C). Serum CK activity, a muscular damage marker, decreased with Ex-AM5 supplementation among four groups (Figure 2D). Trend analysis revealed that AM treatment had a significant dose-dependent effect on increasing blood glucose level (*p* < 0.001) and decreasing serum levels of lactate and ammonia and CK (*p* < 0.001).

### 2.5. Effect of AM Supplementation on Hepatic and Muscle Glycogen Levels

**Figure 3:**
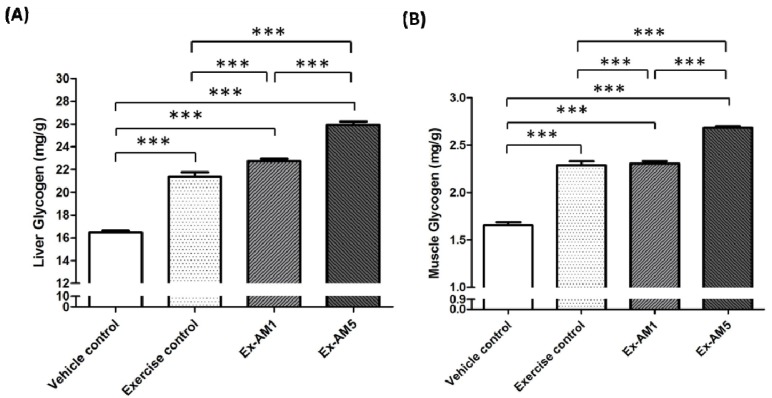
Effect of AM supplementation on levels of hepatic glycogen (**A**) and muscle glycogen (**B**). Data are mean ± SEM of *n* = 10 mice in each group by one-way ANOVA. *** *p* < 0.001.

Hepatic and muscle glycogen levels increased with exercise control, Ex-AM1 and Ex-AM5 treatment (Figure 3). In addition, the trend analysis revealed that AM treatment had a significant dose-dependent effect on increasing hepatic and muscle glycogen levels (*p* < 0.001).

### 2.6. Effect of AM Supplementation on Biochemical Analyses at the End of the Experiment

**Figure 4:**
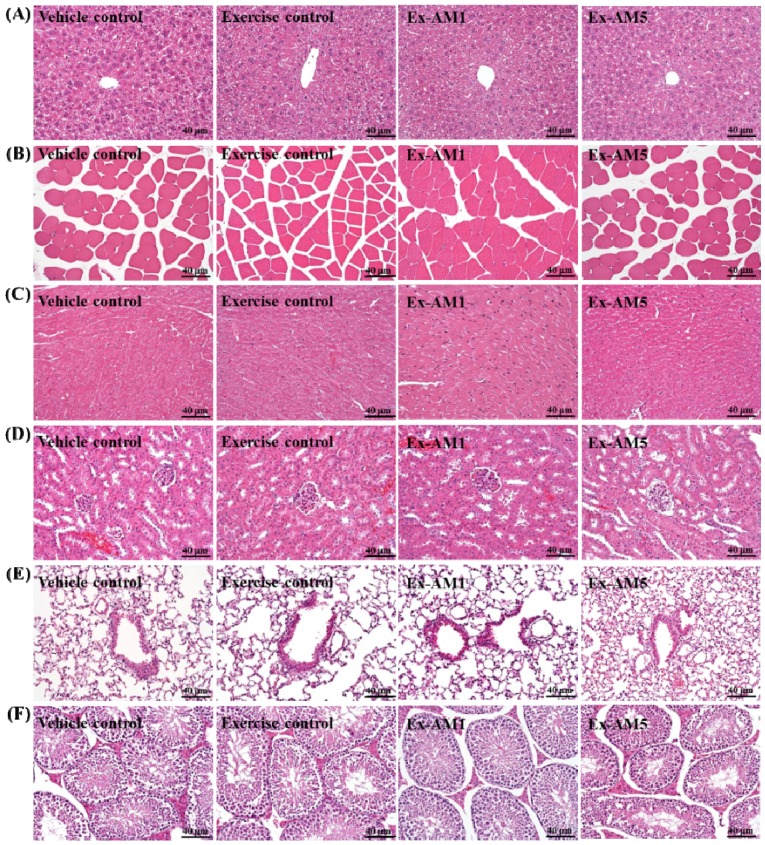
Effect of AM supplementation on morphology of liver (**A**), skeletal muscle (**B**), heart (**C**), kidney (**D**), lungs (**E**), and testes (**F**) tissues. Specimens were photographed under a light microscope. (H&E stain, magnification: ×200, Scale bar, 40 μm).

We examined whether AM treatment for 6 weeks could have negative effects on other biochemical markers in healthy mice. We examined the liver- and kidney-related biochemical parameters and major organs including liver, skeletal muscles, heart, kidney, lungs, and testes according to histopathological examinations in AM-treated mice (Table 2 and Figure 4). We found no indication of a deleterious effect with AM treatment. With exercise training and continuous AM supplementation for 6 weeks, triglycerides level was significantly decreased by about 44% and 40% (*p* < 0.05) for the Ex-AM1 and Ex-AM5 groups, respectively, as compared with the vehicle control. AM may enhance the effect of exercise to reduce hyperlipidemia.

**Table 2:** Biochemical analysis of the AM treatment groups at the end of the experiment.

Parameter	Vehicle Control	Exercise Control	Ex-AM1	Ex-AM5
AST (U/L)	62.90 ± 3.17	68.90 ± 3.78	60.70 ± 2.93	59.00 ± 1.97
ALT (U/L)	42.10 ± 2.84 ^a^	54.30 ± 2.34 ^b^	39.20 ± 1.79 ^a^	46.30 ± 1.93 ^a,b^
ALP (U/L)	48.80 ± 3.22	63.40 ± 5.80	54.80 ± 3.59	59.20 ± 3.49
LDH (U/L)	301.10 ± 19.06	273.00 ± 23.09	254.70 ± 17.29	293.10 ± 15.41
Albumin (g/dL)	3.56 ± 0.08	3.76 ± 0.06	3.59 ± 0.05	3.73 ± 0.06
TBIL (μg/dL)	0.19 ± 0.03	0.22 ± 0.03	0.23 ± 0.03	0.22 ± 0.02
TP (g/dL)	4.73 ± 0.06	4.63 ± 0.06	4.56 ± 0.05	4.58 ± 0.05
BUN (mg/dL)	23.43 ± 0.77	23.07 ± 1.00	20.18 ± 0.57	23.21 ± 0.46
Creatinine (mg/dL)	0.13 ± 0.01	0.12 ± 0.01	0.11 ± 0.00	0.12 ± 0.01
UA (mg/dL)	1.43 ± 0.11 ^a^	0.83 ± 0.05 ^b^	1.33 ± 0.07 ^a^	1.18 ± 0.09 ^a,b^
TG (mg/dL)	228.00 ± 21.03 ^a^	184.40 ± 20.34 ^a,b^	126.70 ± 5.74 ^b^	136.60 ± 10.02 ^b^
TC (mg/dL)	110.60 ± 4.17	104.40 ± 4.45	112.80 ± 4.68	107.90 ± 4.13
Glucose (mg/dL)	179.80 ± 6.03	182.30 ± 6.73	181.00 ± 4.40	177.20 ± 4.56

Data are mean ± SEM for n = 10 mice in each group. Data in the same row with different superscript letters (a and b) differ significantly, *p* < 0.05, by one-way ANOVA. AST, aspartate aminotransferase; ALT, alanine aminotransferase; ALP, alkaline phosphatase; LDH, lactate dehydrogenase; TBIL, total bilirubin; TP, total protein; BUN, blood urea nitrogen; UA, uric acid; TG, triacylglycerol; TC, total cholesterol.

### 2.7. Effect of AM Supplementation on Histological Examinations at the End of the Experiment

As shown in Figure 4, the four groups did not differ in histological observations of liver, muscle, heart, kidney, lung, and testis.

### 2.8. Discussion

Previously, the effect of AM on exercise-induced accumulation of products of metabolism and physical toxicity has been unclear. In this study, we compared the fatigue-alleviating effects of two doses of AM as well as vehicle and exercise control on endurance in exercised and weight-loading mice. We found that: (1) Exercise training combined with AM supplementation increased endurance with exercise and increased hepatic and muscle glycogen content; (2) AM reduced exercise-induced accumulation of byproducts such as blood lactate and ammonia by acute exercise challenge; and (3) daily AM administration for 6 weeks had no toxic effects according to biochemical parameters and histopathological examination. Thus, AM may have ergogenic and anti-fatigue functions.

*Astragalus* polysaccharides are the main active constituents of AM. The *Astragalus* polysaccharide were found to have a positive effect on skeletal muscle for glucose homeostasis through stimulation of protein kinase B (PKB)/glucose transporter 4 (GLUT4) pathways [33]. It has been demonstrated that skeletal muscle 2-deoxyglucose uptake during muscle contractions is directly related to muscle GLUT-4 protein content [34] and GLUT-4-mediated muscle glucose transport does not limit exercise-stimulated muscle glucose uptake [35]. One previous study reported that *Astragalus* polysaccharide increased the number of GLUT4 transporters at the muscle cell surface [33]. In our study, exercise with AM supplement significant increased exercise performance, and AM treatment had a significant dose-dependent effect on increasing blood glucose after a 15-min swimming test without weight-loading. These results indicate that AM may enhance muscle glucose uptake during exercise and continue AM supplement could prolong the time of exercise. 

Serum level of CK is an important clinical biomarker of muscle damage, muscular dystrophy, severe muscle breakdown, myocardial infarction, autoimmune myositides and acute renal failure. High-intensity exercise challenge could physically or chemically cause tissue damage and muscular cell necrosis [36]. Furthermore, reactive oxygen species (ROSs), like lactate anion and protons, have been suggested to be implicated in oxidative skeletal muscle fatigue. It is reported that ROS alter such transport systems as potassium transport and thus contribute to the onset of fatigue [37]. Under oxidative stress-induced cellular injuries, the cell membrane integrity can be damaged, and cytosolic enzymes will leak out into the serum. Those enzymes, including lactate dehydrogenase (LDH), CK, myoglobin, aspartate aminotransferase (AST), alanine aminotransferase (ALT), *etc.*, can be parameters indicating tissue injury under high-intensity exercise challenge [36]. Astragalosides is another bioactive components of AM and a well antioxidant to protect exercise-induced oxidative stress [38,39]. In our study, serum CK activity was significantly lower by 40.7% with Ex-AM5 treatment than the exercise control (*p* < 0.001). These results indicated that AM supplementation could stabilize membranes and attenuate muscle damage during exercise.

Energy storage and supply is another important factor related to exercise performance. With energy expenditure during exercise, physical fatigue is mainly caused by energy consumption and deficiency [40]. Catabolized fat and carbohydrates are considered the main sources of energy during exercise in skeletal muscles, and glycogen is the predominant source of glycolysis for energy production. Therefore, glycogen storage directly affects exercise ability [41]. In our study, liver glycogen content was significantly higher, by 1.06- and 1.21-fold (all *p* < 0.001), with Ex-AM1 and Ex-AM5, respectively, than exercise control. In addition, muscle glycogen content was significantly higher by 1.01- and 1.17-fold (all *p* < 0.001) with Ex-AM1 and Ex-AM5, respectively, than exercise control. The glycogen-sparing effect of AM could provide an important survival advantage *in situ*ations requiring extended periods of prolonged exercise endurance because glycogen depletion is associated with physical exhaustion, and slower utilization of glycogen results in improved exercise endurance. As one of the sources of blood glucose, liver glycogen plays an important role in controlling the availability of cellular energy. It is possible that AM may have promoted glycogenolysis restraint and/or gluconeogenesis.

The levels of serum glucose, lactate, ammonia, and glutamine are known to serve as indicators of accumulated fatigue and stress caused by exercise [42]. Blood lactate is the glycolysis product of carbohydrate under anaerobic conditions, and glycolysis is the main energy source for short term intensive exercise. Because the accumulation of blood lactate causes fatigue during physical exercise, rapid removal of lactate is beneficial to relieving fatigue [43]. Ammonia, a metabolite of proteins and amino acids, was linked to fatigue [44]. An increase in ammonia in response to exercise can be managed by the use of glutamine and/or carbohydrates that interfere with ammonia metabolism [45]. The increase in ammonia level is related to both peripheral and central fatigue during exercise [23]. In our study, serum ammonia levels were significantly lower by 12.7% and 22.3% with Ex-AM1 and Ex-AM5, respectively, than the exercise control (*p* < 0.001). Lactate levels were significantly lower by 25.6% (*p* < 0.05) with Ex-AM5 than exercise control. This result suggests that AM supplementation may reduce exercise-induced byproducts accumulate and alleviate physical fatigue. 

Together, our results suggest that AM supplementation may ameliorate exercise-induced oxidative stress, stimulate blood circulation, improving the transport efficiency to nutritional minerals and assisting excretion and the elimination of the by-products of metabolism, and thereby against physical fatigue.

## 3. Experimental

### 3.1. Experiment Design

The AM used for supplementation in the study was purchased from Sun Ten Pharmaceutical Co., Biotechnology Ltd. (New Taipei, Taiwan). Male ICR strain mice (4 weeks old) grown under specific pathogen free conditions were purchased from BioLASCO (Yi-Lan, Taiwan). One week of acclimation to the environment and diet was allowed before the experiment began. All animals were fed a standard laboratory diet (No. 5001; PMI Nutrition International, Brentwood, MO, USA) and housed at room temperature (23 ± 1 °C), with 50%–60% humidity and lighting (lights on from 06:00 to 18:00). The Institutional Animal Care and Use Committee (IACUC) of National Taiwan Sport University approved all animal experiments in this study, and the study conformed to the guidelines of protocol IACUC-10206 approved by the IACUC ethics committee.

The recommended use of AM for humans is about 3 g per one intake with a normal diet. The mouse AM dose (0.615 g/kg) used in this study was converted from a human equivalent dose (HED) based on body surface area by the following formula from the US Food and Drug Administration: assuming a human weight of 60 kg, the HED for 3 (g) ÷ 60 (kg) = 0.05 × 12.3 = a mouse dose of 0.615 g/kg; the conversion coefficient 12.3 was used to account for differences in body surface area between a mouse and a human as described in our recent study [46].

All animals were randomly assigned to four groups (*n* = 10 per group) for swim exercise training or exercise with AM supplement treatment: (1) sedentary control and vehicle treatment (vehicle control); (2) exercise training with vehicle treatment (exercise control); and (3) exercise training with 0.615 g/kg AM (Ex-AM1) or (4) 3.075 g/kg AM (Ex-AM5). The vehicle group received the same volume of solution equivalent to individual body weight. Both the vehicle and AM were given orally to each animal for 6 weeks.

### 3.2. Swimming Exercise Training

Animals in the exercise control and Ex-AM1 and Ex-AM5 groups underwent an intensive aerobic swim training program adapted from our recent study with some modifications [46]. They were placed in a plastic container (65 cm high, 40 cm diameter) with 20-cm tap water depth maintained at 28 ± 1°C. They trained 30 min on the first day, 45 min on the second day, then 60 min/day, 5 days/week. The swim training was maintained for 1 h from weeks 2 to 6. After the first week, the swim training consisted of 5 weekly sessions of 60 min of forced swimming with a 1% loading of body weight at week 2. From weeks 3 to 4, animals underwent a 2% loading of body weight training protocol. At the fifth and sixth week, the swimming load was up to 3% of body weight, which consisted of 5 weekly swim sessions for 60 min each. Body weight was measured weekly, and the load was estimated and increased accordingly.

### 3.3. Exhaustion Swimming Exercise Test

Swim to exhaustion exercise test involved constant loads corresponding to 5% of body weight to evaluate endurance. The swimming exercise was carried out in a round tank (65 cm high, 40 cm diameter), filled with water to 45 cm depth and maintained at a temperature of 28 ± 1 °C. To avoid circadian variations in physical activity, swimming exercise was performed between 07:00 and 14:00, when minimal variation in endurance capacity was confirmed in mice [47]. The endurance of each mouse was recorded as the time from beginning swimming to exhaustion, which was determined by observing loss of coordinated movements and failure to return to the surface within 7 s. Times floating, struggling, and making necessary movements were considered in the swimming duration until exhaustion and possible drowning.

### 3.4. Forelimb Grip Strength

A low-force testing system (Model-RX-5, Aikoh Engineering, Nagoya, Japan) was used to measure forelimb grip strength of mice undergoing vehicle, exercise, and AM treatments. The amount of tensile force was measured by use of a force transducer equipped with a mental bar (2 mm diameter and 7.5 cm long) for each mouse in each group. As described in our previous studies [31,48,49], we grasped the mouse by the base of the tail and lowered it vertically toward the bar. The mouse was pulled slightly backwards by the tail while the two paws (forelimbs) grasped the bar, which triggered a “counter pull.” This grip strength meter recorded the grasping force in grams. Forelimb grip strength testing was performed after consecutive administration of the vehicle of AM for 6 weeks and 1 h after the last treatment. The maximal force (in grams) recorded by the counter-pull of mice forelimbs was used as grip strength.

### 3.5. Determination of Blood Biochemical Variables

The effects of AM on serum lactate, ammonia, and glucose levels, and CK activity were evaluated post-exercise. At 1 h after the last administration, mice underwent a 15-min swimming test without loading. After the swim exercise, blood samples were immediately collected from the submandibular duct of pretreated mice and centrifuged at 1,500 *×g* and 4 °C for 10 min for serum preparation. Clinical biochemical assessment was determined by use of an autoanalyzer (Hitachi 7060, Hitachi, Tokyo, Japan).

### 3.6. Tissue Glycogen Determination

Because liver and skeletal muscles are the 2 major tissues for glycogen deposition, we investigate whether glycogen contents of these 2 target tissues could increase with AM administration. Mice underwent treatment for 6 weeks and then were killed 1 h after the last treatment administration. The muscle and liver were excised and weighed for a glycogen content analysis. The muscle and hepatic glycogen levels were measured as described in our previous studies [31,48,49]. For each mouse, 60 mg muscle and liver tissue was finely cut, weighed and homogenized in 0.3 mL cold 10% perchloric acid. After centrifugation for 15 min with 15,000 *×g* at 4 °C, the supernatant was carefully decanted and kept on ice for analysis. A standard glycogen (Sigma, Linkou Dist., New Taipei City, Taiwan) or tissue extract, 30 μL, was added to 96-well microplates, and iodine-potassium iodide reagent, 200 μL, was added to each well for binding iodine to glycogen. An amber-brown compound developed immediately after the reaction. Absorbance was measured at wavelength 460 nm with use of an ELISA reader (Tecan Infinite M200, Tecan Austria, Austria) after the material rested for 10 min.

### 3.7. Histological Staining of Tissues

Fresh liver, skeletal muscles, heart, kidney, lungs, and testes tissues were collected and fixed in 10% formalin after mice were killed. Tissues were embedded in paraffin and cut into 4-μm thick slices for morphological and pathological evaluation as we described previously [31,48,49]. Tissue sections were stained with hematoxylin and eosin (H&E) and examined under a light microscope equipped with a CCD camera (BX-51, Olympus, Tokyo, Japan) by a clinical pathologist.

### 3.8. Analysis of Astragalus Membranaceus by HPLC/CAD

To confirm the quality of AM, we analysed the main chemical constituents of AM. The quantitative analysis of AM was confirmed using standard astragalosides I, II, III, and IV (ChromaDex, Irvine, CA, USA) diluted with methanol and then sonicated for 30 min. The mixed standard was further diluted as necessary to create a calibration curve. AM was extracted using methanol and sonicated for 30 min, and was subsequently equilibrated at room temperature. An aliquot of each sample solution was then filtered using a 0.45-μm PTFE syringe filter; the filtrate was collected in an HPLC vial for analysis. The AM component was determined to contain 1.455 mg/g of total Astragaloside (Table 3).

**Table 3:** The Astragaloside compounds in *A. membranaceus* (AM).

	*A. membranaceus* (mg/g)
Astragaloside I	1.02
Astragaloside II	0.24
Astragaloside III	BRL
Astragaloside IV	0.195
Total Astragalosides	1.455

Astragalosides analysis by HPLC/CAD. Chromatographic condition: spectra were obtained by scanning UV-Vis. A Phenomenex Kinetix C_18_ column (150 × 4.6 mm, 2.6 μm, 100 Å) was used. The flow rate was set at 0.9 mL/min, injection volume was 5 μL, and temperature was set at 40 °C. The CAD detection was used corona aerosol discharge detector. BRL, compound detected below reporting limit.

### 3.9. Statistical Analysis

All data are expressed as the mean ± SEM. Statistical differences among groups were analyzed by one-way ANOVA and the Cochran-Armitage test for dose-effect trend analysis with SPSS 14.0 (SPSS, Chicago, IL, USA). In case of significant *F* ratios, Scheffe *post-hoc* tests were used to determine differences. Statistical significance was set at *p* < 0.05.

## 4. Conclusions

*A. membranaceus* (AM) has anti-fatigue activity by decreasing serum lactate and ammonia levels and increasing liver and muscle glycogen deposition, thereby promoting exercise performance in mice. Although the detailed anti-fatigue mechanisms of AM remain to be elucidated, this study provides science-based evidence to support traditional claims of anti-fatigue results with AM treatment and suggests a use for AM as an ergogenic and anti-fatigue agent.
